# An Autotetraploid Linkage Map of Rose (*Rosa hybrida*) Validated Using the Strawberry (*Fragaria vesca*) Genome Sequence

**DOI:** 10.1371/journal.pone.0020463

**Published:** 2011-05-27

**Authors:** Oron Gar, Daniel J. Sargent, Ching-Jung Tsai, Tzili Pleban, Gil Shalev, David H. Byrne, Dani Zamir

**Affiliations:** 1 The Robert H. Smith Institute of Plant Sciences and Genetics in Agriculture, The Hebrew University of Jerusalem, Rehovot, Israel; 2 Malling Research (EMR), Kent, United Kingdom; 3 Department of Horticultural Sciences, Texas A&M University, College Station, Texas, United States of America; The University of Maryland, United States of America

## Abstract

Polyploidy is a pivotal process in plant evolution as it increase gene redundancy and morphological intricacy but due to the complexity of polysomic inheritance we have only few genetic maps of autopolyploid organisms. A robust mapping framework is particularly important in polyploid crop species, rose included (2*n* = 4*x* = 28), where the objective is to study multiallelic interactions that control traits of value for plant breeding. From a cross between the garden, peach red and fragrant cultivar Fragrant Cloud (FC) and a cut-rose yellow cultivar Golden Gate (GG), we generated an autotetraploid GGFC mapping population consisting of 132 individuals. For the map we used 128 sequence-based markers, 141 AFLP, 86 SSR and three morphological markers. Seven linkage groups were resolved for FC (Total 632 cM) and GG (616 cM) which were validated by markers that segregated in both parents as well as the diploid integrated consensus map.

The release of the *Fragaria vesca* genome, which also belongs to the Rosoideae, allowed us to place 70 rose sequenced markers on the seven strawberry pseudo-chromosomes. Synteny between *Rosa* and *Fragaria* was high with an estimated four major translocations and six inversions required to place the 17 non-collinear markers in the same order. Based on a verified linear order of the rose markers, we could further partition each of the parents into its four homologous groups, thus providing an essential framework to aid the sequencing of an autotetraploid genome.

## Introduction

The theory and methodology for the construction of genetic maps in diploid species is well established, whilst mapping in autopolyploids lags behind [1], [2]. Polyploidization has played a major role in plant evolution by increasing gene redundancy and morphological complexity [3], [4], [5], [6], [7], [8]. As a result, polyploid species are often more adaptable and show increased tolerance to different environmental conditions [6], [9], [10]. Many crop species such as alfalfa, sugarcane, potato, sweet potato, tea and rose [11], [12] amongst others, carry multiple copies of the same genome and are classified as autopolyploid.

Genetic segregation in autopolyploids is a reflection of meiosis with a combination of bivalent and multivalent pairing with multiple alleles per locus [13]. Adding to the complexity, multivalent pairing can lead to a unique situation in which the two chromatids originating from the same chromosome may be present together in the same gamete, giving exceptional progeny termed “double reduction” [14]. The complex segregation patterns in the progeny of autopolyploid crosses and the large number of genotypic groups that need to be resolved make it a challenge to construct autopolyploid linkage maps. In practice, sibling genotyping is used to determine the parental genotypes according to the segregation ratio of each marker genotyped, which allows an inference of marker dosage in the parental genotype to be made. The segregation ratio is determined from the ratio of offspring exhibiting the marker to those that do not [15]. Single-dose markers, also called simplex markers, are present with the allelic conformation (Aaaa), whereas double dose markers, also known as duplex markers, have the genotype (AAaa); triplex markers, the genotype (AAAa) and quadriplex markers, the genotype (AAAA). Nulliplex (aaaa) describes a parental genotype where the marker is absent [13] (Fig. S1).

Different theories and methods have been developed to overcome the difficulties associated with autopolyploid mapping. Initially, genetic maps were constructed for cultivated polyploid plant species according to linkage maps of diploid relatives, such as in potato [16]. Later Wu et al [17] proposed a general method for autotetraploid mapping using only simplex markers that was implemented in sugarcane [18]. The autopolyploid linkage map that published by Al-Janabi et al [19] in sugarcane was the first map constructed directly from a complex polyploid species without the aid of either diploid relatives or a classical linkage map. Subsequently da Silva et al [20] integrated this map with the simplex-based map of Sobral et al [18] and added duplex and triplex markers showing it is possible to use multi-dose markers if a framework linkage map was available. More recently, sophisticated theories and methods have been developed for autopolyploid mapping by relying on the dosage identification for each marker and assigning chromosomes to homologous sets [1], [21]- an issue unique to autopolyploids [1]. Statistical methods and theories using Markov Chain models were recently implemented by both Leach et al [2] for tetrasomic multilocus analysis, and by Baker et al [15] for allocating marker dosage in autopolyploids species.

“TetraploidMap” [22] is the only publically available software application that has been developed for autotetraploid mapping. The software performs calculations based on the simplest situation that can arise from tetrasomic inheritance, namely random pairing of four homologous chromosomes to give two pairs of bivalents at meiosis. In practice, many departures from this simple situation can occur, in particular: multivalent pairings and double reduction; lack of complete homology between chromosomes and hence departures from random pairing; and distorted segregation due to differential fertility and viability [23]. In spite of these considerations, the suitability of this software for linkage and QTL analysis in potato and alfalfa has been demonstrated [23], [24], [25] which led us to try to implement it for the construction of the autotetraploid maps of *Rosa hybrida*.

Due to its ubiquitous and long-standing popularity, the rose has become the most economically-important ornamental crop worldwide for cut flowers, garden ornamentals and potted flowering plants. Roses belong to the Rosaceae family and are therefore related to important fruit crops including strawberry, apple, peach and cherry. Wild rose species range from diploid to octoploid forms, whereas cultivated roses which are perennial are mostly highly heterozygous autotetraploids (2*n* = 4*x* = 28) with a small genome estimated at about 550 Mb (0.57 pg/1C) [26]. The major mapping efforts in the genus, recently reviewed by Spiller et al [27], have been concentrated at the diploid level, using a double pseudo testcross strategy (Fig. S2) which is suited for allogamous species with strong inbreeding depression [28]. Four different mapping populations allowed the construction of an integrated consensus map (ICM) consisting of 597 markers distributed across seven linkage groups, with an overall length of 530 cM [27]. The ICM facilitated the resolution of genes and QTL affecting flower morphology (double flowers, petal number, flower color and white-striped flowers), plant morphology (prickles and growth vigor), fertility (self incompatibility), flowering (days to flowering and recurrent blooming), scent metabolites, and disease resistance (black-spot and powdery mildew). However, as rose breeding is mainly performed at the tetraploid level, it is important to develop a tetraploid map that could be used for mapping QTL of value for rose improvement and for use in the development of tools and germplasm for marker assisted breeding [2], [24].

**Figure 1:**
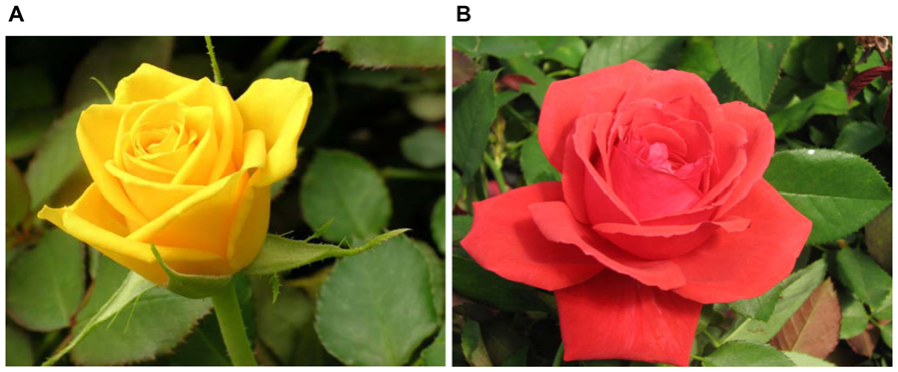
The *Rosa hybrida* L. cultivars used as parents of the segregating population (GGFC). **A**. *Rosa hybrida* cv. Golden Gate (GG) is a modern cut-flower cultivar, containing carotenoids that are responsible for its yellow color, with only faint odor and long vase life. **B**. *Rosa hybrida* cv. Fragrant Cloud (FC) is an old garden cultivar with large fragrant flowers, short vase life and peach red petals color due to the presence of anthocyanins (Short movie presenting the vase life behavior of these cultivars is available at http://www.youtube.com/watch?v=odOp92TK5Xg - the movie composed of pictures that were taken every 60 minutes with total time lapse of 15 days).

Ten years ago we initiated a rose genomics project aimed at identifying genes for fragrance. Two rose varieties were selected as the basis of the research: “Golden Gate” (GG) and “Fragrant Cloud” (FC) (Fig. 1). The large peach red FC flowers possess a strong scent, accumulate anthocyanins, and have a short vase life, whereas the medium yellow flowers of GG accumulate carotenoids, have a long vase life, and lack a distinct odor [29]. It is interesting to note that although GG is nearly odorless to humans, insects are highly attracted to its scent [30]. The high level of scent polymorphism between these varieties allowed us to create an annotated petal EST database of ∼2100 unique genes from both cultivars and to identify, and complement in bacteria, several scent-related genes [29].

To date, no available genome sequence exists for the *Rosa* genus with which to validate the positions of markers located to autotetraploid linkage maps. However, *Rosa* belongs to the Rosoideae subfamily of the Rosaceae [31], and is well-supported as the closest sister taxon to a clade containing the genus *Fragaria*. Recently, Villanova et al and Illa et al [32], [33] reported a high degree of conservation of synteny between the distantly-related Rosaceous genera *Fragaria*, *Malus* and *Prunus* and demonstrated a large number of conserved syntenic blocks, some of which spanned whole chromosomes between genera. The genome of the diploid strawberry species *F. vesca* (FvH4) (2*n* = 2*x* = 14) was recently sequenced to 39× coverage and anchored to the diploid *Fragaria* genetic map [34]. The close genetic relationship between *Fragaria* and *Rosa* suggests that the FvH4 sequence could be used as a reference for which to validate markers mapped in the *Rosa* autotetraploid mapping progeny and elucidate the level of synteny between the *Rosa* and *Fragaria* genomes.

To further characterize the genetic basis of the differences between the FC and GG rose cultivated varieties, we have developed two autotetraploid maps for *Rosa hybrida* using large numbers of transferrable sequenced-based markers for a progeny of 132 siblings with the software application “TetraploidMap”. We have validated the map through a comparison of linkage group marker placement and marker order on each of the parental linkage maps, and a comparison to the integrated consensus map of diploid *Rosa*. To characterize the genetic relationships between the genomes of *Rosa* and *Fragaria*, both members of the Rosoideae sub-family of the Rosaceae, and to further validate marker order, we compared the positions of orthologous markers mapped to the autotetraploid map to their positions on the seven pseudo-chromosomes of the diploid *Fragaria* genome sequence.

## Results

### Marker segregation

The strategy for constructing an autotetraploid rose genetic map was to use a combination of conserved, sequence-characterized markers (RFLP and CAPS), to allow comparisons with sequenced Rosaceous genomes and other marker types (AFLP, SSR and morphological) to increase marker density. Markers were divided into uni-parental markers, showing heterozygosity (simplex or duplex dosage) in a single parent, and bi-parental markers, showing heterozygosity in both parents. The coding nomenclature method of marker segregation types, segregation ratios and scoring method is presented in Table 1. All the segregation types were assigned to markers after a χ^2^ test with a null hypothesis according to their accepted segregation ratio (significance level of the χ^2^ test P>0.001) to determine the parental genotypes. All markers were binary scored as “1”-present/“0”-absent.

**Table 1:** The markers used to construct the rose map.

	Uni-parental markers				Bi-parental markers				
Parent map	FC		GG		FC & GG				Total
**Scoring method**	Dominant	Dominant	Dominant	Dominant	Dominant	Dominant	Dominant	Codominant	
**Parents genotype (FC X GG)**	Aaaa X aaaa	AAaa X aaaa	aaaa X Aaaa	aaaa X AAaa	Aaaa X Aaaa	Aaaa X AAaa / AAaa X Aaaa	AAaa X AAaa	multiple alleles	
**Segregation rate**	01∶01	01∶05	01∶01	01∶05	01∶03	01∶11	01∶35		
**Segregation group type**	1	2	3	4	5	6	7	8	
									
**AFLP**	63	10	23	17	28	14			**155**
**RFLP**	5	4	8	4	6	1	1	9	**38**
**SSR**	20	7	19	8	9	13	4	35	**115**
**CAPS**	48	21	38	15	3	1		11	**137**
**Morphological**	2	2							**4**
**Total**	**138**	**44**	**88**	**44**	**46**	**29**	**5**	**55**	**449**

Markers grouped by marker type and segregation ratios that were assigned after a χ^2^ test. Markers from segregation type 6 (1∶11) and in other cases where it was impossible to determine the parents genotypes were not used in the mapping.

### Marker systems

Of the ∼700 markers that we used to screen the GGFC population 449 polymorphic markers were scored. Out of those markers, 358 (80%) that could be associated with the parental genotypes were used for map construction.

#### AFLP

Using seven AFLP primer pairs, a total of 155 polymorphic markers were scored on the mapping population (Table 1). From those we were able to map, eighty-six (55%) segregated as simplex (1∶1); 27 (17.5%) segregate as duplex (5∶1); 28 (18%) double-simplex (3∶1) and 14 (9%) of the markers showed segregation of (11∶1) and were not used in map construction (Table S2).

#### RFLP

RFLP analysis was conducted mostly for candidate genes that may be associated with the production of fragrance compounds, as well as genes that could potentially affect flower morphology. Using 38 RFLP markers (Table 1), we scored 63 polymorphic loci ( = alleles). Ten of the markers each hybridized to single loci in the rose genome while the remaining 28 belonged to small gene families and showed multiple banding. Nine (23.6%) were scored as codominant; 17 (44.7%) were scored as dominant, and for the remaining 12 markers the determination of the parental genotypes was not possible. Thus we mapped 26 RFLP markers.

#### SSR

More than 100 SSR primer pairs were previously used in generating the various diploid rose maps [27]. In order to associate the GGFC tetraploid maps with the existing diploid maps we used 63 labeled SSR primers out of those pairs in this study. With them 115 alleles ( = bands) were scored but only for 102 could the parental genotypes be determined (Table 1). For 34% and 7% of these SSRs all the alleles from specific primer pairs were read together as codominant and dominant, respectively. Over the 35 polymorphic SSR loci with codominant segregation, the average number of alleles per locus in both parents was 2.3 of the potential 8 allelic positions in the two autotetraploid parental genotypes.

For the majority of the SSRs, it was impossible to determine the parental genotype when reading all alleles together for specific primer pairs. Thus, in these cases each allele was read separately enabling us to map 44 more alleles ( = markers) giving a total number of 86 mapped SSR markers.

#### CAPS

We generated 323 CAPS markers based on the previously described EST database that was established using the population parents [29] and NCBI rose sequences. Out of those 323 markers, 137 CAPS markers were polymorphic (Table 1). For 100 markers (73%) we were able to determine the parental genotypes. For the remaining 37 markers when the marker was multiallelic each of the alleles amplified was scored separately producing 17 more alleles ( = markers). Thus we were able to map 102 CAPS markers.

#### Morphological

**Figure 2:**
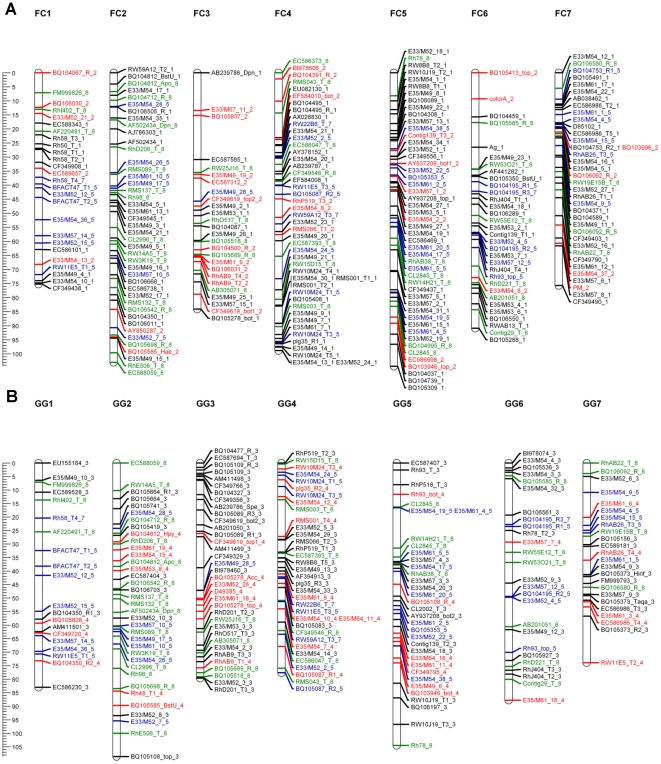
The parental linkage map. Each map consists of seven linkage groups. Map distances are shown in cM as a ruler at the left page margin. Marker names are assigned according to the nomenclature described in Table S1. Each color represents a different segregation ratio (black for 1∶1, red for 5∶1, blue for 3∶1 and green for codominant markers). **A**. Map of Fragrant Cloud (FC). **B**. Map of Golden Gate (GG).

We scored four phenotypic qualitative traits and then translated the phenotypic data into present/absent data. Integration with the marker data enabled us to treat each trait as a single marker, which allowed us to map the genomic region controlling each trait. Three traits were placed on the map: anther color (*Ag*) and flower color (*Color_A*) mapped to FC LG 6, and powdery mildew (*PM*) resistance mapped to FC LG 7 (Fig. 2). None of the siblings had yellow flower color (*Color_Y*) like the parent GG, suggesting monogenic tetrasomic inheritance in which the yellow flower color is recessive.

### Map construction

Map construction was performed using “TetraploidMap for windows”[25]. ^2^χ test was performed for all 449 markers in order to determine the parental genotypes on the basis of the segregation in their offspring. For 403 markers (dominant and codominant) the parental genotype could be determined, with a significance threshold larger than 0.001 (^2^χ test) (Table S2) and were used to construct the maps. Despite passing the χ^2^ test, the parental genotypes of 29 (7%) markers that segregated 11∶1 could not be determined, and thus were excluded from the procedure. A further 16 (4%) of the markers showed distorted linkage patterns and thus these markers were excluded from the analyses.

Cluster analysis of all markers where parental genotypes were identifiable generated seven linkage groups corresponding to the basic chromosome number of the rose. To reveal possible discrepancies in the grouping, we performed a pre-ordering analysis by combining a two-point analysis with an initial-run and the ripple search. This facilitated the identification of ∼3% of the markers that were wrongly placed, for which we re-checked their recombination frequency and LOD score values compared to the other markers in the group. If such markers did not fit in the particular linkage group they were excluded or moved manually to a different linkage group that generated lower recombination frequency and higher LOD scores. In the next step we reran the ordering analysis with the simulated annealing algorithm that explores the best possible orders and the maps were drawn. A total of 358 markers were placed on the maps (Table 2). For FC (Fig. 2A), the map length was 632 cM, with 259 markers with an average distance between markers of 2.4 cM and a largest gap of 14 cM (Table 2). The map of GG (Fig. 2B) covered 616 cM, with 210 markers positioned, an average distance between markers of 2.9 cM and a largest gap of 17 cM (Table 2).

**Table 2:** Distribution of markers on parental maps (GG and FC) and linkage group statistics.

Linkage Group	AFLP	RFLP	CAPS	SSR	Morphological	Total	Common Markers	Length (cM)	Average Distance (cM)
FC 1	8	1	7	10	0	**26**	11	75	2.88
FC 2	16	4	14	9	0	**43**	22	103	2.40
FC 3	9	2	11	4	0	**26**	12	84	3.23
FC 4	15	5	10	16	0	**46**	22	99	2.15
FC 5	26	1	15	7	0	**49**	18	104	2.12
FC 6	9	4	9	8	2	**32**	14	91	2.84
FC 7	16	5	9	6	1	**37**	12	76	2.05
**Total (FC)**	**99**	**22**	**75**	**60**	**3**	**259**	**111**	**632**	**2.44**
GG1	5	2	7	6	0	**20**	11	83	4.15
GG2	11	4	12	9	0	**36**	22	109	3.03
GG3	6	5	17	7	0	**35**	12	80	2.29
GG4	12	5	2	16	0	**35**	22	78	2.23
GG5	15	1	9	9	0	**34**	18	104	3.06
GG6	9	4	6	7	0	**26**	14	88	3.38
GG7	9	3	5	7	0	**24**	12	74	3.08
**Total (GG)**	**67**	**24**	**58**	**61**	**0**	**210**	**111**	**616**	**2.93**

### Integrated map and comparison to diploid data

**Figure 3:**
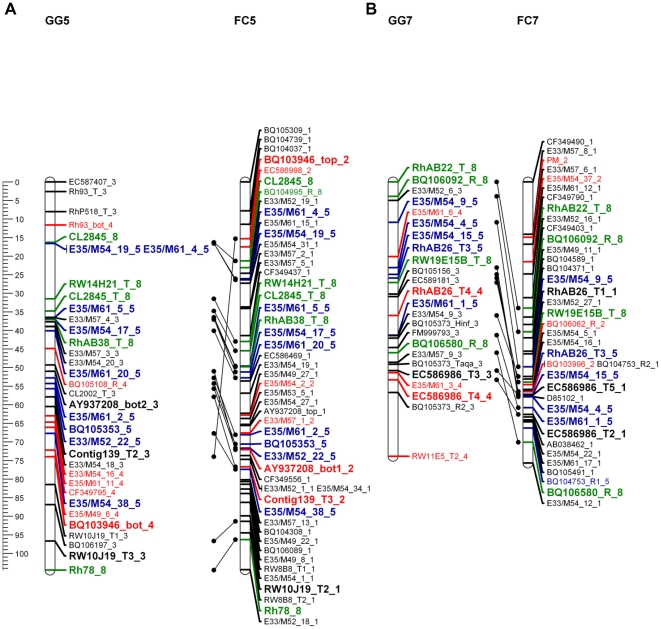
The linear order of common markers conserved in both parental maps. Each linkage group name contains the parental name and the linkage group number. Map distances are shown in cM as a ruler at the left page margin. Marker names indicated according to the nomenclature described in Table S1 **.** Each color represents a different segregation ratio (black for 1∶1, red for 5∶1, blue for 3∶1 and green for codominant markers). Common markers are indicated in bold and larger font. **A.** Linkage group 5. **B**. Linkage group 7. The remaining linkage groups are presented in Fig. S5.

**Figure 4:**
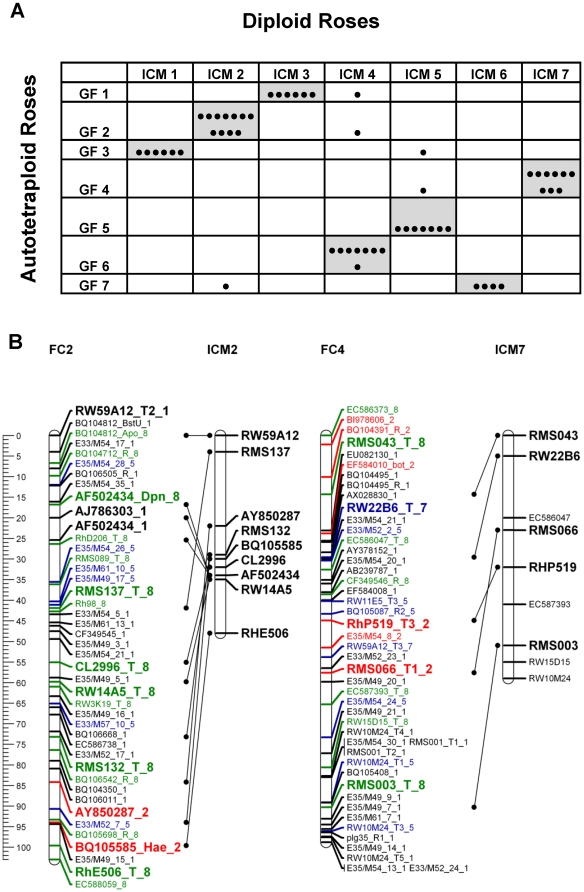
A comparison of the tetraploid GGFC maps with the diploid ICM. **A**. Number of markers of each diploid ICM linkage group that correspond to the autotetraploid linkage groups of the GGFC maps. Each marker is indicated by a black dot. Cells that contain more than one marker are noted with a grey background. **B**. The linear order of conserved markers between the FC map and ICM maps. Results are shown for FC2-ICM2 and FC4-ICM7. Map distances are shown in cM as a ruler at the left page margin. The linkage group numbers appear above each group. FC marker names are indicated according to the nomenclature described in Table S1. Each color represents a different segregation ratio (black for 1∶1, red for 5∶1, blue for 3∶1 and green for codominant markers). The ICM marker names and positions are given according to Spiller et al [27]. Black lines connecting the common markers. The markers that present on the ICM but not connected to the FC map are corresponding to the GG map.

To validate the GGFC map we compared common markers on the FC and GG maps as well as common markers that were analyzed in diploid rose maps [27]. The accessibility of bi-parental markers, and especially those that were codominant, facilitated the identification of homologous linkage groups and the integration of both parental maps was done manually (Fig. 3; Fig. S5). Among the 111 common markers, the linear order was maintained for 88 (80%). Moreover, more than 95% of these markers appeared in the same linkage groups in both parents. Because the order of the majority of common markers was similar in both maps, we conclude that the positions of markers on the integrated map are reliable. A comparative analysis of the GGFC map with the recently published ICM for diploid rose [27] revealed that 51 of the 56 common markers (91%) were located on the same linkage group in both maps (Fig. 4). Additionally the total length of the ICM covers 85% of both GG and FC maps suggesting that their genome coverage is similar.

### Synteny of *Rosa* and *Fragaria*

**Figure 5:**
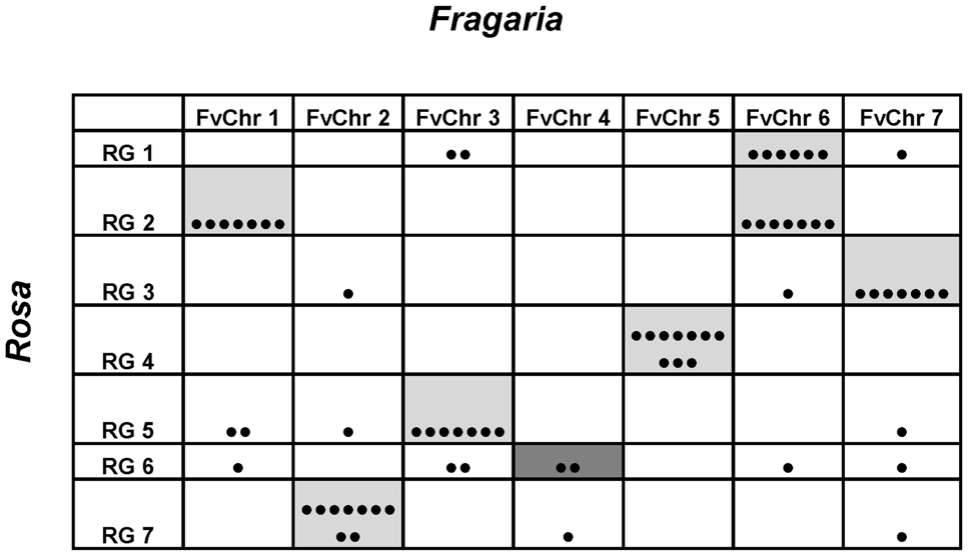
*Rosa* FC-*Fragaria* pseudo-chromosome comparison. The number of markers of each autotetraploid FC linkage group (RG) that correspond to the pseudo-chromosomes of the *Fragaria* FvH4 reference sequence (FvChr). Each marker is indicated by a black dot. Cells that contain more than three markers are shaded with a grey background. The tentative relationship between RG6 and FvChr4 (two markers) is shown with a dark grey background.

A total of 70 EST markers were used for comparison to the *Fragaria* FvH4 genome sequence assembly. The markers comprised those mapped to a single locus on the FvChr map (*F. vesca* Chromosomes map), corresponding to a single unambiguous position on the FvH4 genome sequence. The distribution of the 70 markers across the seven linkage groups of *Rosa* was relatively even (Fig. 5), ranging from 13 markers on RG2 (synonymous to Fragrant cloud linkage group), to seven markers on RG6 (average 10 markers per linkage group). Average marker densities ranged from 3.54 cM/marker on RG7 to 10.75 on RG6 (6.92 cM/marker average over the seven *Rosa* linkage groups). The map distance covered by the 70 markers was 484.71 cM, 77% of the coverage of the FC map constructed with all markers. Distribution of the 70 markers across the *Fragaria* pseudo-chromosomes was similar to *Rosa*, with a maximum of 14 markers on FvChr 6, 11 markers on each of FvChr 2, 3, 5 and 7, nine markers on FvChr 1 and three markers on FvChr 4.The total physical distance covered by the markers was 139.14 Mbp, 70% of the total genome sequence scaffolds anchored to the seven FvH4 pseudo-chromosomes.

**Figure 6:**
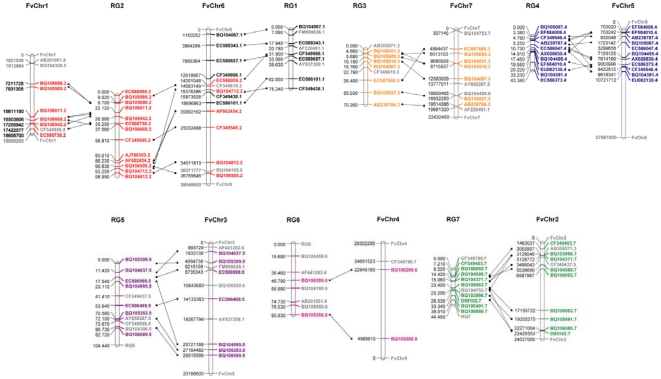
Comparison between the *Rosa* FC linkage map and the pseudo-chromosomes of *Fragaria* FvH4 genome sequence. Each group contains the map positions of the 70 orthologous markers used for comparison. Lines between *Rosa* linkage groups (RG) and *Fragaria* pseudo-chromosomes (FvChr) indicate marker positions within syntenic blocks. Map distances are given in cM, pseudo-chromosome positions are given in nucleotides. Marker names are given with the suffix according to the *Rosa* linkage group on which they are mapped. Markers common to syntenic blocks are given in the color of the *Rosa* linkage group; non-syntenic markers are given in grey. Delimiters defining the ends of the *Fragaria* pseudo-chromosomes and where necessary the *Rosa* linkage groups are given in grey with the pseudo-chromosome/linkage group name.

The chromosomes, to which 54 markers (77%) were located, were conserved between *Rosa* and *Fragaria*. With the exception of FvChr 4, to which just three *Rosa* ESTs were located, all *Fragaria* pseudo-chromosomes contained sufficient markers to infer syntenic relationships between *Rosa* and *Fragaria* (Fig. 5). Conservation of macro-synteny was high between all *Rosa* linkage groups and *Fragaria* chromosomes, with the majority of markers on each linkage group in *Rosa* locating to a single *Fragaria* pseudo-chromosome. *Rosa* linkage group 3 corresponded to FvChr 7, RG4 to FvChr 5, RG5 to FvChr 3, RG7 to FvChr 2, whilst *Rosa* linkage groups 1 and 2 corresponded to *Fragaria* chromosomes 1 and 6. A tentative relationship between RG6 and FvChr 4 was inferred, although group FvChr 4 contained only two markers mapped to RG6, and the markers on RG6 displayed the least conservation of synteny with *Fragaria* (Fig. 6). Collinearity of markers between *Rosa* and *Fragaria* was high with an estimated four major translocations and six inversions required to place the 17 non-collinear markers in the same order on each genome. The most collinear groups were RG3 and FvChr 7, and RG5 and FvChr 3, whilst the least conserved were RG4 and FvChr 5. Markers that had been mapped to RG6 were distributed between five *Fragaria* chromosomes, with synteny observed between just two mapped markers on RG6 and FvChr 4.

### Partitioning into homologous sets

**Figure 7:**
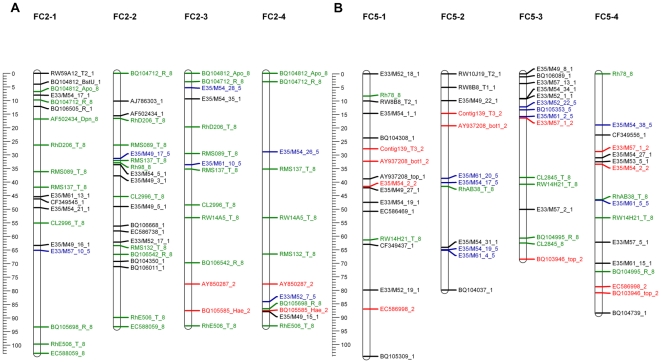
Two of the Fragrant Cloud (FC) linkage groups showing the four homologous chromosomes (1–4). Map distances are shown in cM as a ruler at the left page margin. Marker names indicated according to the nomenclature described in Table S1 **.** Each color represents a different segregation ratio (black for 1∶1, red for 5∶1, blue for 3∶1 and green for codominant markers). **A**. Linkage group 2. **B**. Linkage group 5.

The genetic map of an autopolyploid species has two components: linkage groups and homologous sets. After validating the first component we were able to tackle the second. Using the pairwise results, recombination frequency, LOD score and the coding of the simplex markers provided by the “TetraploidMap” software [25], we were able to manually determine the phase of each of the ordered markers enabling each of the seven linkage groups to be separated into four homologous chromosomes (Fig. 7). Importantly, this procedure is only available in autopolyploid designated software as homologous sets are unique to these species.

## Discussion

In cut rose breeding, a common experience is that in a cross between two roses with classical flowers (http://www.youtube.com/watch?v=odOp92TK5Xg), the probability of recovering a progeny of high quality, at least as high as the parents is ∼0.00001. One contributor to the complex genetics in roses is the strong inbreeding depression where often weak and albino progeny are derived. Thus homozygous lines are not available and therefore, we opted to construct an autotetraploid linkage map based on a cross of two unrelated heterozygous parents – Fragrant Cloud (FC) which is a garden cultivar and Golden Gate (GG) a cut rose – to create an F1 segregating population using a double pseudo testcross strategy [28] (Fig. S2). In the last 10 years, by exploiting the EST database previously established using these varieties, we were able to design and map 128 sequence-based markers (CAPS and RFLP) which are scarce in previously published rose genetic maps [27], [35], [36], [37], [38]. The sequences of these markers also permitted comparisons between the GGFC maps and sequenced rosaceous genomes, including that of *F. vesca*. Combining the EST markers with AFLP, SSR and morphological markers allowed marker density to be increased. Using these data, and employing the only available software suitable for the construction of genetic linkage maps of autotetraploid species, we have constructed an autotetraploid linkage map for rose.

The core issue in the construction of a derived map especially for an autopolyploid species is its validation. In order to confirm the fit of the GGFC map we initially used the basic character of the double pseudo testcross strategy that provides individual maps for each of the parents. Using both dominant and codominant markers with different dosage allowed us to compare and integrate the two parental maps (Fig. 3; Fig. S5); over 95% of the common markers group to the same LG in both parents. Moreover, an average of 16 markers per LG (80% of the total number of the common markers) showed a consistent collinear order between the parental maps, indicating that map construction and marker ordering was reliable. Comparing these results to the linkage maps in other autotetraploid species [23], [24] shows that such high marker consistency between the parents is unique to this rose work.

The comparison between the GGFC map and the ICM for diploid rose [27] was performed using 56 common markers, of which 91% (4–11 common markers per linkage group) were located to the same linkage group in both maps. Moreover, in some linkage groups the collinearity of marker order was well conserved between the ploidy levels (up to 91% in FC LG2 in Fig. 4A). For the marker ordering it is noticeable that although it showed consistency it could be improved via an increase in marker density. Nevertheless, the similarity between the diploid and the autotetraploid rose maps is consistent with studies in other genera within the Rosaceae, such as between the diploid and the allooctoploid strawberry [39], [40].

### Synteny between *Rosa* and *Fragaria* genomes

A comparative analysis was performed using 70 EST-based markers mapped to the *Rosa* FC linkage map and physically-located to the FvH4 genome sequence [34] that fulfilled the criteria set out in the materials and methods. The markers represent good coverage of both the *Rosa* linkage map (77%) and the *Fragaria* genome sequence (70%). Average density of orthologous markers used for comparison was similar to that achieved in a comparative study between *Malus, Fragaria* and *Prunus* [33], and comparable to genomic comparisons based on linkage maps in species of other families [41], [42], [43].

Using a similar number of markers (71) to those used in this study, Villanova et al [32] revealed a high degree of synteny between the diploid *Fragaria* and *Prunus* linkage maps, showing that markers mapping to a single *Prunus* linkage group were located on just one or two *Fragaria* linkage groups. Similar patterns of synteny were revealed in a comparison of the reference maps of *Prunus* and *Fragaria* to the *Malus* × *domestica* cultivar “Golden Delicious” (MpGD) genome sequence [33]. Their study revealed large macro-syntenic blocks between the genomes of the three genera and validated the marker relationships revealed by Villanova et al [32], demonstrating that predictions about synteny of related genera can be made with a high degree of accuracy and precision using the numbers of markers we have employed in this investigation.

*Rosa* and *Fragaria* belong to the Rosoideae subfamily of the Rosaceae [31] with the genus *Rosa* well-supported as the closest sister clade to that containing *Fragaria* and *Potentilla*. Thus *Fragaria* and *Rosa* are closely related genetically, and this is reflected in the conservation of synteny between the structures of their respective genomes (Fig. 5), where most of the markers that mapped to a single linkage group in *Rosa* located on one *Fragaria* pseudo-chromosome, consistent with highly conserved syntenic genome blocks observed throughout the Rosaceae. Markers that were not located within syntenic regions may represent paralogous loci or translocation events that have occurred since the two genera diverged from a common ancestor, but this could not be determined with the density of common markers analysed in this investigation. Observed collinearity was high, with an estimated four translocations and six inversions required to put all syntenic markers in the same order on both genomes (Fig. 6). The comparisons presented here are extending the knowledge of comparative biology of the Rosaceae to a new clade- the Rosoideae, and will help elucidate the patterns of evolution that have occurred since the subfamily diverged from its common ancestor with the Spireaeoideae.

Our results indicate that there is sufficient synteny between the genomes of *Rosa* and *Fragaria* to allow the information from the FvH4 genome sequence of strawberry to inform genetics and genomics studies in *Rosa*. Recently, it has been demonstrated that a trait found in both *Rosa* and diploid *F. vesca*, perpetual blooming, or *semperflorens* is governed in both species by a mutation in the same homologous gene (Fabrice Foucher, personal communication [Unpublished]). Moreover, as shown here, the morphological trait flower color peach red (*Color_A*) which mapped to the end of FC LG6 is similar to the *B* gene in *Prunus* (almond/peach petal color) which mapped to the end of LG1 in the more distantly-related *Prunus* reference map [44], a region shown to be syntenic to FvChr4 [32], [33], which we have demonstrated here to be syntenic to FC LG6 (Fig. 6). These examples showing the potential benefit of our work to “translational genomics” studies in Rosaceae. Thus, it makes sense in the next phase to compare the multitude of QTL for common morphological and biochemical traits that were resolved for the rose GGFC with *Fragaria* and other Rosaceae.

### Concluding remarks

The map for the cultivated autotetraploid rose that we present here, is a step towards understanding how multiple alleles interact genetically to control plant phenotypes. It was previously noted that the problem of constructing genetic maps in autopolyploids is twofold; loci must be ordered along individual chromosomes, and the chromosomes must be assigned to homologous groups [1]. The first problem can be solved with better ordering algorithms, and is also a common problem in the construction of diploid linkage maps, the latter problem however, is unique to autopolyploids. In this investigation, we were able to overcome both obstacles (Fig. 3 and Fig. S5 for ordering; Fig. 7 for homologous group). Moreover, by mapping sequence-based markers we have demonstrated highly conserved synteny between *Rosa* and *Fragaria* (Fig. 5; Fig. 6). Full mapping of the 28 chromosomes of the autotetraploid rose is an essential step towards QTL analysis. In the future we will present a large scale trait and QTL analysis for more than 400 ontology defined characters that were repeatedly measured on the GGFC population.

The advent of next-generation sequencing technologies has made whole genome shot-gun sequencing (WGSS) affordable and accessible to the entire biological research community and has thus enabled genome sequence data to be generated for virtually any species under investigation. However, the high degree of homology between the closely related genomes in autopolyploid species, coupled with an equally high degree of heterozygosity within those sub-genomes precludes the assembly of WGSS for autotetraploid *Rosa* species [45]. Here we present the development of a linkage map for *Rosa hybrida* from which we have characterised all 28 linkage groups. These maps, when populated with additional markers, can provide a framework for the development of a map-based resource to enable the sequencing, assembly and anchoring of a genome sequence for tetraploid rose. Additionally, as phenotyping is the rate limiting factor for discovery, the phenotypic traits measured over the past 10 years on the GGFC population make their parents “Golden Gate” and “Fragrant Cloud” attractive candidates for autotetraploid sequencing.

## Materials and Methods

### Plant material

A double pseudo testcross population (GGFC) of 132 individuals was generated from the crosses conducted in 2001 and 2002 between the parents “Golden Gate”® (GG) bred by W. Kordes' Söhne, and “Fragrant Cloud”® (FC) bred by RosenWelt Tantau (Fig. 1). Progeny of the cross were grown in pots filled with a peat:volcanic gravel mixture (1∶1, v/v) in a greenhouse under controlled temperature (28/20°C day/night) and a natural photoperiod. Genomic DNA of each of the GGFC genotypes was extracted according to Roche et al [46] and used for map construction.

### Molecular markers

#### AFLP markers

Analyses were conducted by KeyGene N.V as describe by Vos et al [47] using the restriction enzyme combination of *Eco*R1 (E) / *Mse*I (M). Selective amplification was carried out with the primers: E33/M52, E35/M49, E35/M54, E33/M54, E33/M57, E35/M53 and E35/M61.

#### RFLP markers

The RFLP probes were generated using the sequenced clones constructed by Guterman et al [29]. A total of 20 µg genomic DNA from parental varieties and their progeny were loaded and separated on 1% agarose gels after digestion with one of four restriction enzymes; *DraI* ***,*** *Hind*ΙΙΙ, *Eco*RI (New England Biolabs Inc., USA) *MvaI* (F. Hoffmann-La Roche Ltd., Switzerland), and blotted to positively charge nylon membrane Hybond XL (Amersham Biosciences, Sweden). Probes were radioactively labeled using the random primers method with Dctp^32^ [48]. Electrophoresis, Southern blotting, hybridization and nick-translation of probes was performed according to Bernatzky and Tanksley [49].

#### SSR markers

A total of 63 SSR primers were analyzed. The PCR reaction mixture contained 1 µl DNA (10 ng), 0.5 µl HEX, TET, FAM or NED fluorescently labeled forward primer, 0.5 µl reverse primer, 2.5 µl DNase/RNase- free water, 5 µl GoTaq Green Master Mix (Promega Corporation, Madison, WI) and 0.5 µl MgCl_2_ (25 mM stock solution). The PCR reactions were performed in a TECHNE TC-412 thermal cycler (Bibby Scientific Limited, UK) programmed for one step of denaturation at 94°C for 3 min. followed by 30 cycles of denaturation at 94°C for 30 sec., primer annealing at 55°C for 45 sec. and primer extension at 72°C for 1 min. A final extension step was carried out at 72°C for 7 min. and then held at 4°C. Multiplex fluorescently labeled PCR products (1 µl) generated from various SSR primers were added to 8.5 µl Hi-Di Formamide and 0.5 µl ROX400. This mixture was run through the capillary sequencer, ABI 3100 (Life Technologies Corporation, Carlsbad, CA). The DNA peaks (sizes) separated on ABI 3100 were analyzed with the GeneScan and Genotyper software (Life Technologies Corporation, Carlsbad, CA). The SSR names coding and primers sequences is according to Spiller et al [27].

#### CAPS (Cleaved Amplified Polymorphic Sequences) markers

These markers were mainly generated from the EST databases construct by Guterman et al [29]. PCR primers (Table S2) were designed with Primer3 software (http://frodo.wi.mit.edu/primer3) using the default settings.

A total of 19 previously characterized genes and markers [36], [50], [51], [52] were also used as CAPS markers. Standard PCR reactions were performed with 50 ng of template DNA in a 25 µl PCR reaction containing 1x PCR buffer [53], 5 pmol of each primer, 2.5 mM dNTPs, and 2.5 U Taq polymerase (Gene Choice Inc., USA). PCR were conducted with a 90 sec. initial denaturation at 94°C, 35 cycles of 20 sec. denaturation at 94°C, 30 sec. annealing at a primer-specific annealing temperature, and 75 sec/kb product elongation at 72°C, followed by a 10 min. final elongation at 72°C. CAPS markers were generated by digestion of PCR products with 5 U of restriction enzyme for 3 hours at temperatures specified by the manufacturers. Polymorphism of the PCR products or digestion products for the CAPS markers were visualized on 3% agarose gel with ethidium bromide according to Sharp et al [54].

All types of markers were scored as “0” (fragment absent) or “1” (fragment present). In the case of codominant (multiallelic) markers each allele was first scored separately (e.g. “0” or “1”) and then as a group to allow analysis of tetrasomic inheritance.

#### Morphological characters

Morphological traits segregating in the progeny include: anther color (Gramene Trait Ontology TO:0000187)- *Ag*; flower color (TO:0000572)- *Color_A* and *Color_Y*; and resistance to Powdery Mildew (TO:0000439)- *PM*.

Anther color phenotypes were determined in two different years (2005 and 2009) by visual inspection in the greenhouse (yellow/anthocyanic; Fig. S3). Yellow anthers are sometimes difficult to distinguish from pale anthocyanic colored anthers. For this reason we scanned the flower organs of the whole population using a Hewlett-Packard scanjet 4400c® and double-checked the anther color on the computer screen. (All photos are available at http://phnserver.phenome-networks.com).

Flower color phenotypes- peach red FC color (yes/no) and yellow GG color (yes/no) were determined in two years (2005 and 2006) by visual inspection in the greenhouse (Fig. 1 for the parental color).

Powdery Mildew disease in roses caused by the fungi *Sphaerotheca pannosa* (Wallr) Lev. var. *rosae* War. is one of the most common disease damaging both greenhouse and open field roses in Israel [55]. Powdery Mildew resistant and susceptible phenotypes were determined in two different years (2009 and 2010) by visual inspection in the greenhouse (“1”-resistance/“0”-susceptible; Fig. S4).

### Map construction and comparisons

To construct a genetic linkage map for each of the parents we used the software “TetraploidMap for Windows” (http://www.bioss.ac.uk/knowledge/tetraploidmap) [22], [25].

Five main steps were employed to construct the linkage maps using “TetraploidMap”.

1) Analysis of single marker segregation (“FINDGENO”) where the most likely dosage for each marker, conditional on the observed parent and offspring phenotypes, was identified, without or with double reduction. 2) Clustering into linkage groups (“CLUSTER”) conducted on each parent for the markers identified as simplex, using the simple matching coefficient that is equivalent to the recombination frequency for simplex coupling linkages. This identified markers that mapped to the same chromosome. All simplex, duplex and multiallelic markers were then analyzed by group average cluster analysis to partition them into LGs, analyzing markers from the two parents separately. 3) Estimation of recombination frequency between all pairs of markers within a linkage group (“TWOPOINT”) where for each LG recombination frequencies and LOD scores were calculated between every pair of markers for all possible phases using the EM algorithm. 4) Ordering, based on the pairwise data (“SIMANNEAL”) where recombination frequencies and LOD scores from the phase with the highest likelihood were used to order the markers. A simulated annealing algorithm was used to identify the order with the minimum value of the weighted least squares criterion and to calculate map distances between the markers [22], [23], [25]. 5) In the final step, the chromosomes were assigned to homologous groups [1]. A panel of pairwise results that shows the most likely phase for any pair of ordered markers, together with their recombination frequency, LOD scores and the coding of the simplex markers enabled manual inference of the phase of the ordered markers [25]. With the phase information, each linkage group was reconstructed into four homologous chromosomes.

Comparisons between the parental maps were performed manually using the common markers. Comparisons between the ICM of diploid rose [27] and the autotetraploid maps were performed manually and were done only for the FC map which had a higher number of mapped markers. Linkage maps, the comparison of the parental maps, homologous chromosomes and the FC map comparison to the ICM diploid map were presented using MAPCHART 2.2 for Windows [56].

### Nomenclature of linkage groups and markers

The complexity of inheritance in autopolyploids leads to a greater number of segregation types in the population siblings than in diploid progenies [57]. Combining five different marker systems to generate marker data, using dominantly and codominantly scored markers, the presence of single to multiple alleles and the occurrence of uni-parental and bi-parental markers, prompted the use of detailed nomenclature to provide as much information as possible for a specific marker in a straight-forward manner on the map figures. The systematic marker nomenclature appears in Table S1. All marker names were composed from three components, describing the “serial number”; molecular type along with the scoring method and segregation ratio.

### Comparative mapping and marker validation between *Rosa* and *Fragaria*

The seven pseudo-chromosomes of the *Fragaria vesca* (FvH4) genome sequence [34], were used to locate sequenced GGFC markers to the *Fragaria* genome. To evaluate the conservation of synteny between *Rosa* and *Fragaria*, 128 sequence-characterized markers from both the FC and GG *Rosa* linkage maps were used as queries for BLASTN, using a cut off E-value of 1e-15. A greater number of markers located to the FC map identified significant matches with orthologous sequences in the *Fragaria* genome sequence assembly, and thus a comparison was made between positions of markers from the FC map to the *Fragaria* pseudo-chromosomes. Markers were considered for comparison only if they mapped to a single discrete position on the FC *Rosa* linkage map, and matched to a single unambiguous position on the *Fragaria* genome, to which no other EST sequences were significantly aligned. A syntenic relationship between two sections of the *Rosa* and *Fragaria* genomes was defined when at least three orthologous markers were present in the same section of both genomes. *Rosa* linkage groups (RG) and *Fragaria* pseudo-chromosomes (FvChr) and links between homologous markers were plotted using MAPCHART 2.2 for Windows [56].

## Supporting Information

Figure S1 **Possible allelic constitutions in autotetraploids.** The loci A–F illustrate the possible genotypes at one locus with two alleles (capital letter represent dominant allele). The terminology monogenic nulliplex, simplex, duplex, triplex and quadriplex describe the dosage of the dominant allele at the loci A, B, D, E and F respectively. Locus H shows codominant allele that contain up to four different alleles.
(TIF)

Figure S2 **Double pseudo testcross strategy compare to classic pure line hybridization. A**. Crossing two heterozygous parents results in a segregating sibling population that can be use for constructing individual maps for each of the parents. **B**. Crossing two homozygous parents (pure lines) results in uniform variety with specific characteristics from either or both parents.
(TIF)

Figure S3 **Anther color (** ***Ag*** **) phenotype scoring.** **A**. Yellow colored filament score as “0”. **B**. Anthocyanic colored filament score as “1”.
(TIF)

Figure S4 **Resistance to Powdery Mildew (** ***PM*** **) phenotype scoring. A**. Scored “0” for susceptible siblings. **B**. Scored “1” for resistant siblings.
(TIF)

Figure S5 **The linear order of the common markers preserved in both parental maps**. Each linkage group name contains the parent name and the linkage group number. Map distances are shown in cM as a ruler at the left page margin. Marker names are indicated according to the nomenclature described in Table S1 **.** Each color represents a different segregation ratio (black for 1∶1, red for 5∶1, blue for 3∶1 and green for codominant markers). The common markers are indicated in bold and larger font. **A**. Linkage group 1. **B**. Linkage group 2. **C**. Linkage group 3. **D**. Linkage group 4. **E**. Linkage group 6.
(TIF)

Table S1 **Nomenclature of the markers which were used to construct the genetic linkage map in autotetraploid roses.** For multiallelic markers that were scored codominantly, when the parental genotype identification failed each of the alleles amplified by the primer pairs were scored dominantly and separately (RFLP (II), SSR (II) and CAPS (II)). The detailed nomenclature makes it possible to infer the marker properties directly from the maps figures.
(XLS)

Table S2 **Characteristics of the 449 polymorphic markers used us in this work.** Marker type, total number of alleles, expected phenotype and genotypes of the parents as determined by “TetraploidMap” software, segregation ratio and the Chi-squared test (χ^2^) its statistical significance (ratio_sig), double reduction coefficient (α) and its statistical significant (DR_sig) are given for each marker. Where possible the marker data also includes blast information, primers, restriction enzyme used and the band sizes for FC and GG.
(XLS)
