# Supplementary figures and images for: An Autotetraploid Linkage Map of Rose (Rosa hybrida) Validated Using the Strawberry (Fragaria vesca) Genome Sequence
Source: PLoS One. 2011 May 27;6(5):e20463. doi: 10.1371/journal.pone.0020463 (PMC3103584; doi:10.1371/journal.pone.0020463)

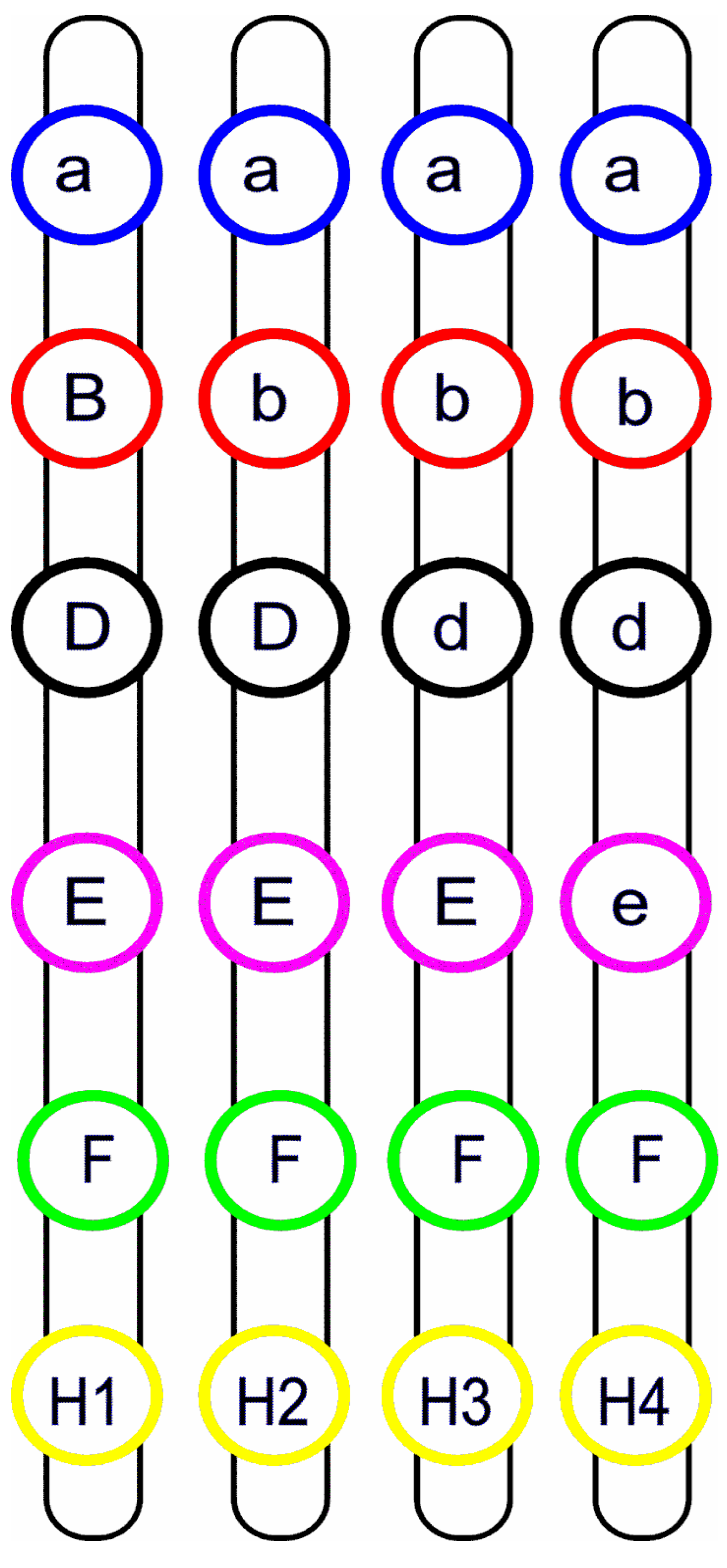

Supplement: Figure S1 — Possible allelic constitutions in autotetraploids. The loci A–F illustrate the possible genotypes at one locus with two alleles (capital letter represent dominant allele). The terminology monogenic nulliplex, simplex, duplex, triplex and quadriplex describe the dosage of the dominant allele at the loci A, B, D, E and F respectively. Locus H shows codominant allele that contain up to four different alleles. (TIF) [file pone.0020463.s001.tif]

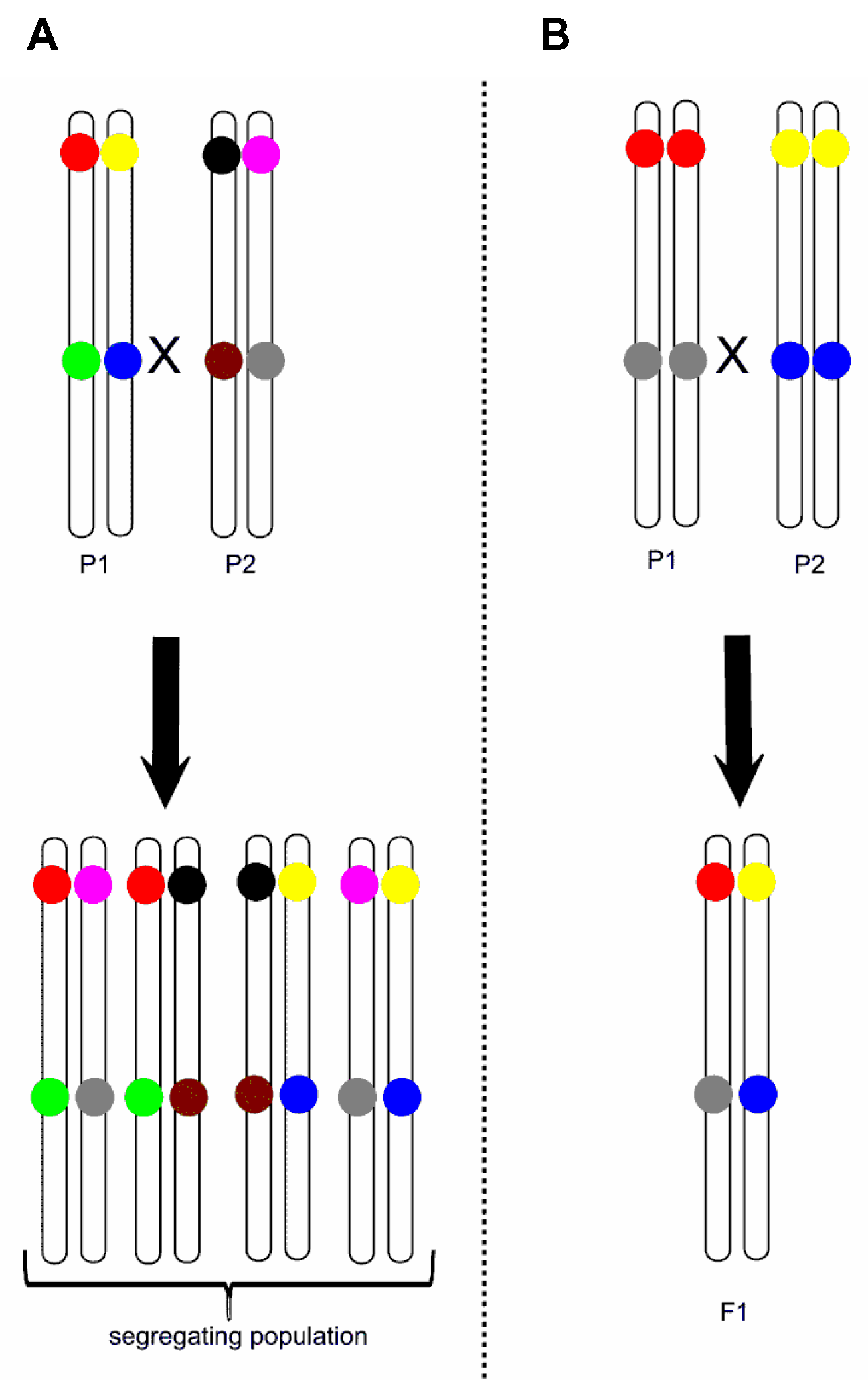

Supplement: Figure S2 — Double pseudo testcross strategy compare to classic pure line hybridization. A. Crossing two heterozygous parents results in a segregating sibling population that can be use for constructing individual maps for each of the parents. B. Crossing two homozygous parents (pure lines) results in uniform variety with specific characteristics from either or both parents. (TIF) [file pone.0020463.s002.tif]

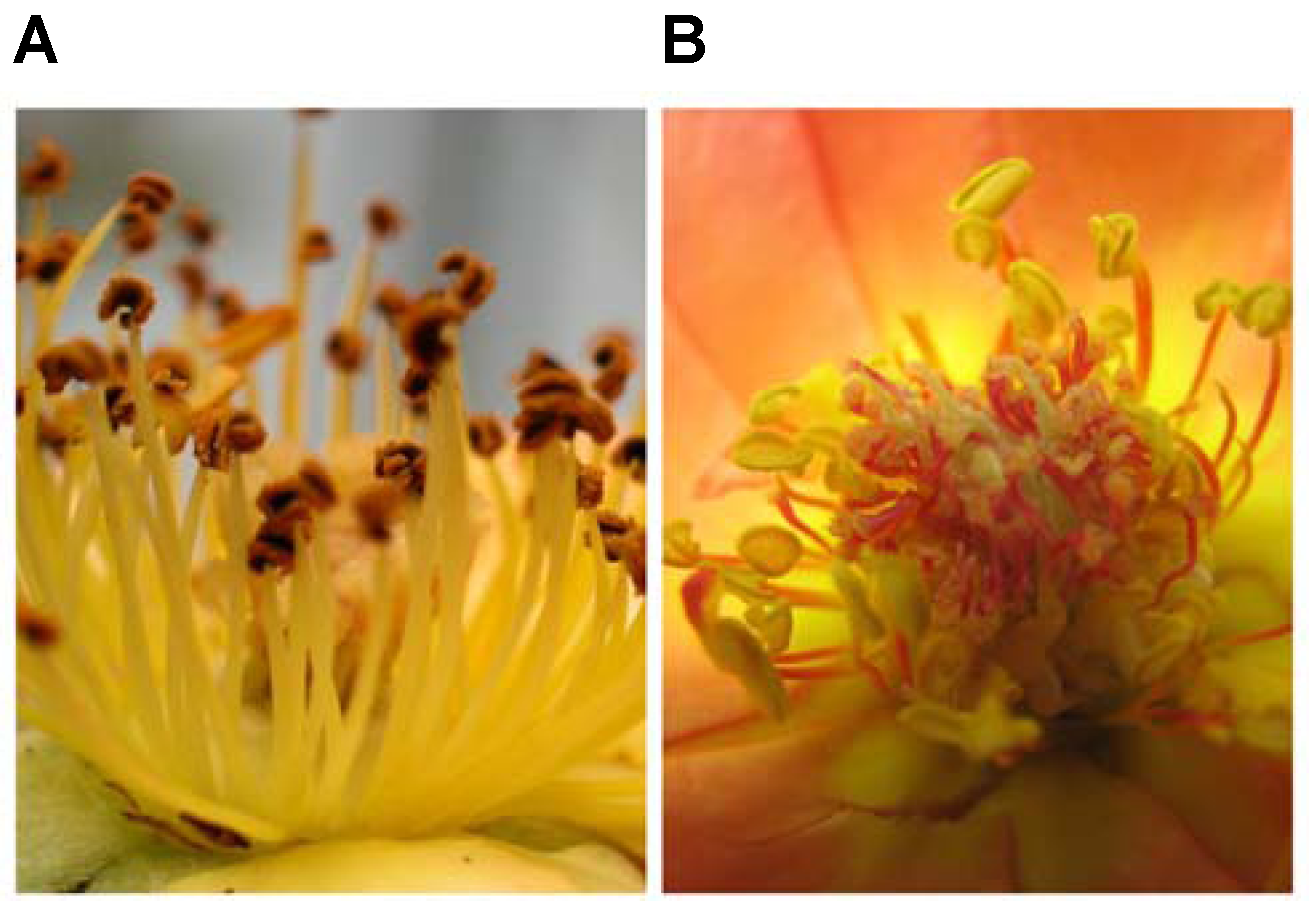

Supplement: Figure S3 — Anther color ( Ag ) phenotype scoring. A. Yellow colored filament score as “0”. B. Anthocyanic colored filament score as “1”. (TIF) [file pone.0020463.s003.tif]

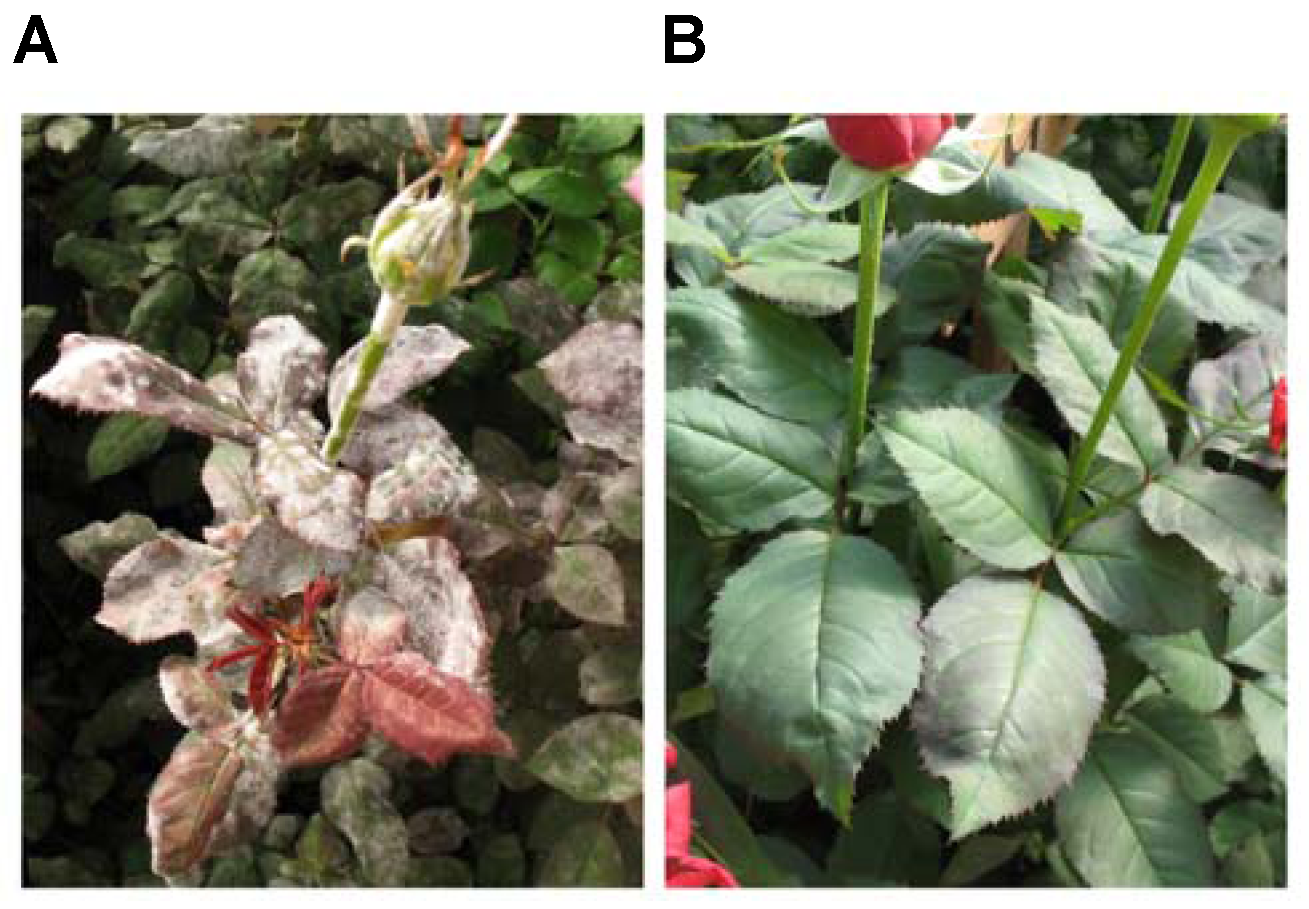

Supplement: Figure S4 — Resistance to Powdery Mildew ( PM ) phenotype scoring. A. Scored “0” for susceptible siblings. B. Scored “1” for resistant siblings. (TIF) [file pone.0020463.s004.tif]

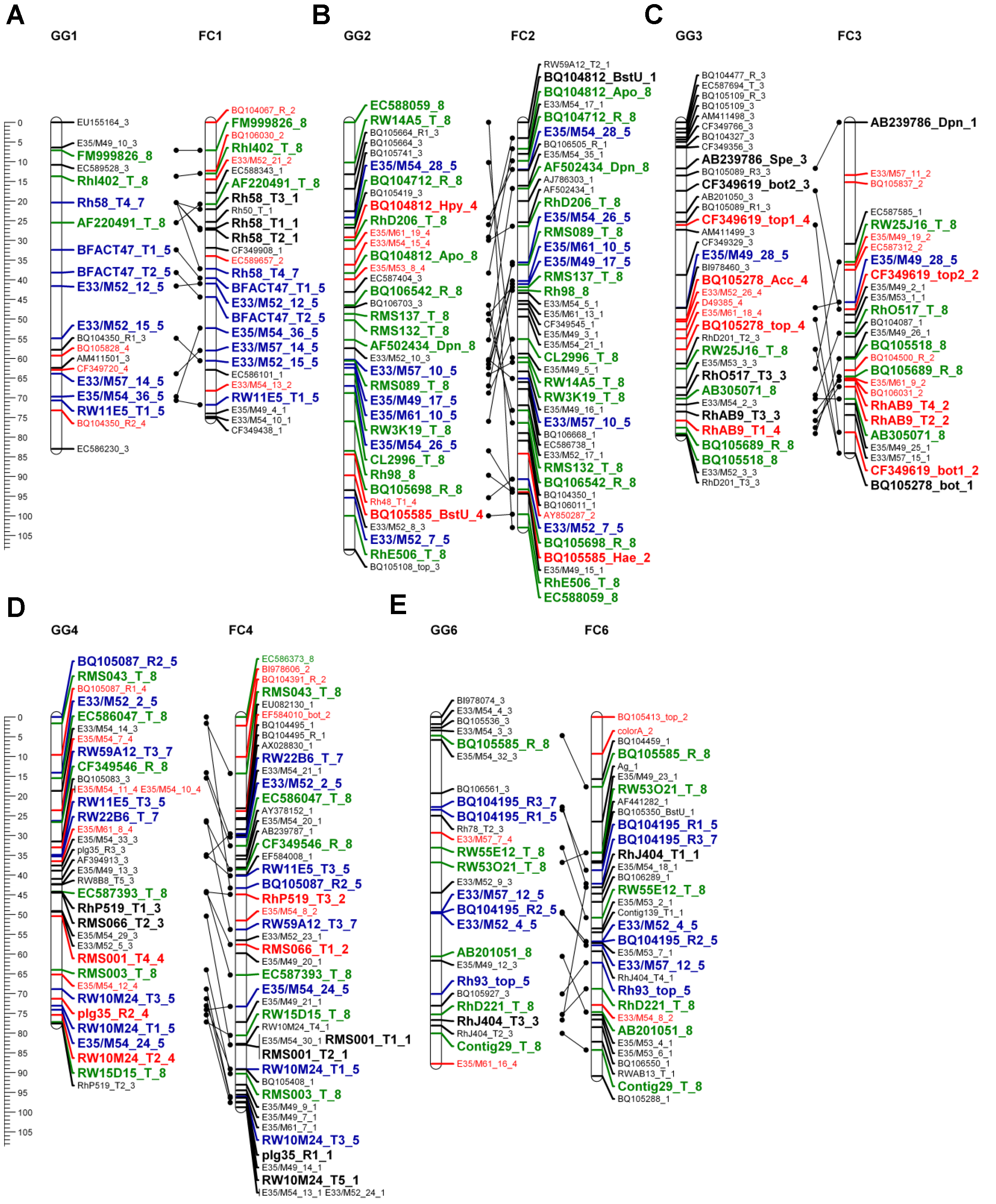

Supplement: Figure S5 — The linear order of the common markers preserved in both parental maps. Each linkage group name contains the parent name and the linkage group number. Map distances are shown in cM as a ruler at the left page margin. Marker names are indicated according to the nomenclature described in Table S1 . Each color represents a different segregation ratio (black for 1∶1, red for 5∶1, blue for 3∶1 and green for codominant markers). The common markers are indicated in bold and larger font. A. Linkage group 1. B. Linkage group 2. C. Linkage group 3. D. Linkage group 4. E. Linkage group 6. (TIF) [file pone.0020463.s005.tif]
